# Role of Infection and Immunity in Bovine Perinatal Mortality: Part 1. Causes and Current Diagnostic Approaches

**DOI:** 10.3390/ani11041033

**Published:** 2021-04-06

**Authors:** John F. Mee, Paulina Jawor, Tadeusz Stefaniak

**Affiliations:** 1 Animal and Bioscience Research Department, Teagasc, Moorepark Research Centre, P61 P302 Fermoy, County Cork, Ireland; 2 Department of Immunology, Pathophysiology and Veterinary Preventive Medicine, Wrocław University of Environmental and Life Sciences, 50-375 Wrocław, Poland; paulina.jawor@upwr.edu.pl (P.J.); tadeusz.stefaniak@upwr.edu.pl (T.S.)

**Keywords:** primary and secondary pathogens, *Neospora:* BVDv, *Bacillus: Leptospira*, perinatal mortality, stillbirth, necropsy, foetus, placenta

## Abstract

**Simple Summary:**

Death of calves before, during and up to 48 h after birth is referred to as bovine perinatal mortality (PM). This is caused mainly by calving problems. However, some cases of PM are caused by infections (pathogenic microbes). Infections include bacteria, viruses, parasites and fungi, and in some cases, multiple pathogens. Diagnosis of infection as a cause of PM can be difficult as one needs to differentiate between exposure to the pathogen, infection by the pathogen and the pathogen causing PM. Most cases of PM attributed to infection are caused by infections acquired in utero. Investigation of infectious PM involves collecting a health history of the dam, the calf and their herd, post-mortem examination of the dead calf and its placenta and collecting appropriate samples for laboratory testing.

**Abstract:**

While non-infectious causes are more commonly diagnosed in cases of bovine perinatal mortality (PM), the proportion caused by infections is highly variable between studies (~5–35%); the reasons for this variation, and possible underestimation, are discussed. The most important pathogen-specific infectious causes of PM are bacteria (in particular, *Bacillus licheniformis* and *Leptospira* spp.), viruses (in particular BVDv) and a parasite (*Neospora caninum*). However, co-infection may occur in a small proportion of cases and in many cases no single pathogen is detected but gross or microscopic lesions of an inflammatory response are identified. Diagnosis is complicated by the criteria required to establish exposure, infection and causation. Additionally, pathogens can be classified as primary or secondary though such differentiation can be arbitrary. The majority of infectious cases of PM are due to in utero infections but postnatal infections (0–2 days) can also cause PM. Diagnosis of infectious PM is based on a systematic investigation of the herd health history and dam and cohort sampling and examination of the perinate and its placenta. Gross and histopathologic examinations and maternal/herd and perinate serology form the basis of current infectious PM investigations.

## 1. Introduction

Perinatal mortality (PM) may be defined as the death of a fullterm (≥260 day) foetus or perinate which dies within 48 h of birth. To be clear, PM encompasses deaths which occur in utero, during parturition and postnatally, hence the descriptor ‘*perinatal*’ meaning around birth. The term postnatal, as used in this manuscript, means within two days of birth. It is an international animal health and welfare issue [1]. Broadly speaking, the causes of PM may be classified as infectious and non-infectious. Non-infectious causes, in particular dystocia (abnormal calving) and asphyxia, are more common than infectious causes (Table 1). In general, infectious causes account for approximately 15% of PM cases, varying widely between studies (~5–35%), (Table 1). Infection commonly ranks as the second or third most common diagnosed cause of PM. However, these data originate in studies where not all calves/organs/tissues are sampled and where the number of pathogens tested for is variable. Thus, it is probable that all studies underestimate the role of infection in PM of calves.

The aims of this review are, to present the state-of-the-art in published knowledge on the infectious causes of bovine PM globally and the current methods of diagnosis. While reference is made to both abortions (termination of gestation prior to 260 d of gestation) and neonatal mortality (calf death after two days of age) where necessary, the focus of this review is on the perinatal period as defined above.

## 2. Infectious Causes of Perinatal Mortality

This section will deal with three aspects of the infectious causes of perinatal mortality: the basic principles underlying such infections, the pathogens causing these infections and the current methods of diagnosis of these pathogens as causes of perinatal mortality. Novel, future diagnostics as well as response to infections are discussed by Jawor et al., [2] in Part 2 of this mini-series.

### 2.1. Principles of Perinatal Infections

Before discussing the individual pathogens and their diagnosis, it is necessary to discuss more basic but equally important aspects of the relationship between the pathogen and the host. These include the types of infectious agents, the occurrence of co-infections, the principles of primary and secondary infections, the profile of in utero versus postnatal (0–2 days) infections, the concepts of exposure, infection and causality, the ‘role’ of infection in PM, the proportion of PM caused by infection and finally, the infectious agents causally linked to perinatal mortality.

#### 2.1.1. Types of Infectious Agents

Bacteria, viruses, parasites and fungi are known to cause PM in calves. Bacterial infections are the most common type of pathogen infection in PM calves, though this can vary widely between studies. For example, studies in the UK [3], Poland [4] and in Denmark [5] reported a low prevalence of stomach content-culture positives in stillborn calves—2, <2 and 0.8%, respectively. However, a much higher percentage of culture positives were recorded in a Finnish study [6] where a significant bacterial isolation was made in 10% of calves which died within 24 h of birth. Some viruses are relatively commonly detected in PM calves (e.g., BVDv), e.g., [7] and on occasion, in epidemics, viruses may be even more commonly detected, e.g., the emergence of novel viruses, for instance, Schmallenberg virus [8]. While most parasites are not detected in PM calves, one particular protozoal parasite, *Neospora caninum*, is the most common pathogen detected in many bovine PM studies [4,9,10]. Fungi are detected in PM calves at a very low rate though they are detected more frequently in studies where both aborted foeti and perinates are combined [11,12]. Thus, bacterial, viral and neospora infections are the most frequently diagnosed types of infections in PM calves.

However, in many cases of PM infection a single (or multiple pathogen) is not detected (or detectable, e.g., autolysis, contaminated, scavenged carcass or incorrect/inadequate sampling, especially absence of the placenta) but compelling gross or microscopic lesions indicative of inflammatory response to a pathogen is detected [13,14]. Thus, describing foetopathogens exclusively does not adequately describe the ‘picture’ of PM infections.

The data in Table 1 refer to both detection of pathogens and of lesions indicative of an inflammatory response to pathogens.

#### 2.1.2. Co-Infection

Although in the majority of infections, a single pathogen is detected, dual infection in a single calf is not uncommon [21,23,24], but reports of multiple co-infections, particularly in multiple foetuses, are uncommon [25]. Diagnosis of multiple infections, after excluding opportunistic agents, suggests a high environmental infectious challenge. This may occur where a common pathogen is endemic in a herd, e.g., *N caninum* or *Coxiella burnettii* and infected calves are co-infected with other/another, often sporadic, pathogen [4,21].

#### 2.1.3. Primary and Secondary Pathogens

Primary pathogens are capable of crossing the intact placenta and causing placentitis [26], foetopathy [27] or luteal regression [28] due to their intrinsic virulence in healthy cows and so often require a low infective dose. Primary pathogens are often associated with outbreaks of PM. An extensive list of examples is shown in Table 2.

Secondary or opportunistic pathogens (now termed pathobionts or facultative pathogens or in some cases, opportunistic emerging pathogens—[29]) are incapable of transplacental infection unless the placenta is damaged, the microflora in the reproductive tract is altered (dysbiosis) or the cow is immuno-compromised (e.g., by prior BVDv infection). Examples are listed in Table 2. They can infect the foeto-placentum once the cervix is open (ascending infection) or from the pregnant cow’s bowel or lungs or a focus of infection, e.g., abscess (haematogenous infection). While these opportunistic agents are usually not considered a primary cause of foetopathy, if they induce a bacteraemia or septicaemic or auto-immune response, or exacerbate a co-infection, they may result in PM [30]. Hence, their isolation is often reported by laboratories as of ‘uncertain significance’. These organisms are part of the normal microbiome of the host and its environment (e.g., poorly preserved forage) and tend not to be contagious. Secondary pathogens are usually associated with sporadic PM [29].

While the lists compiled in Table 2 are generally accepted [3,29,31,32,33,34], they are not unanimously agreed. For example, the following organisms are considered secondary, not primary pathogens by some authors: *Bacillus licheniformis* [35], *Listeria monocytogenes* [3,36], *Salmonella* Dublin [3], *Trueperella pyogenes* [3,33,36] while *Ureaplasma* spp are considered primary, not secondary pathogens by Anderson [31].

Irrespective of whether a pathogen is considered primary or secondary, in order for it to be considered the cause of PM three criteria need to be satisfied: the pathogen is found in heavy or pure growth in the abomasum and/or other tissues, there is an associated inflammatory process in the foetal tissues and/or placenta and other causes of PM can be excluded. The third criterion is important where infection may be a co-morbidity but not the cause of death. For example, where the first two criteria are fulfilled but there is a compelling co-existing cause of death—infection in a calf which suffers a fractured spine during traumotocia. In this case the calf is infected but the probable cause of death is trauma, not infection, though a diagnosis of co-mortality; infection and traumotocia may yield a richer profile of the circumstances surrounding the calf’s death.

#### 2.1.4. Infectious Agents Linked to Perinatal Mortality

The pathogens most commonly detected in perinatal mortality studies are *Neospora caninum* and viruses (e.g., BVD), followed by *Bacillus* spp and *Leptospira,* and, to a much lesser extent, other bacteria and fungi (Table 3). The proportion of *infections* (not cases of PM) attributed to each of these pathogens varies widely between studies (2–100%) primarily due to the limited number of cases detected with infectious agents in many studies. Only three pathogens (*Neospora caninum* [4], viruses (BVD) [22] and *Bacillus* spp. [14]) have been detected in 50% or more of *infected* perinatal mortality cases where a pathogen is recorded. However, given the low proportion of all-cause PM attributed to infection or where infection is detected, individual pathogens are rarely detected in more than 10% of PM cases. The pathogens outlined, alphabetically, hereunder have been most frequently linked to PM in calves. This is not an exhaustive list; see Table 2 and veterinary textbooks for further details.

##### *Bacillus* *licheniformis*

**Figure 1:**
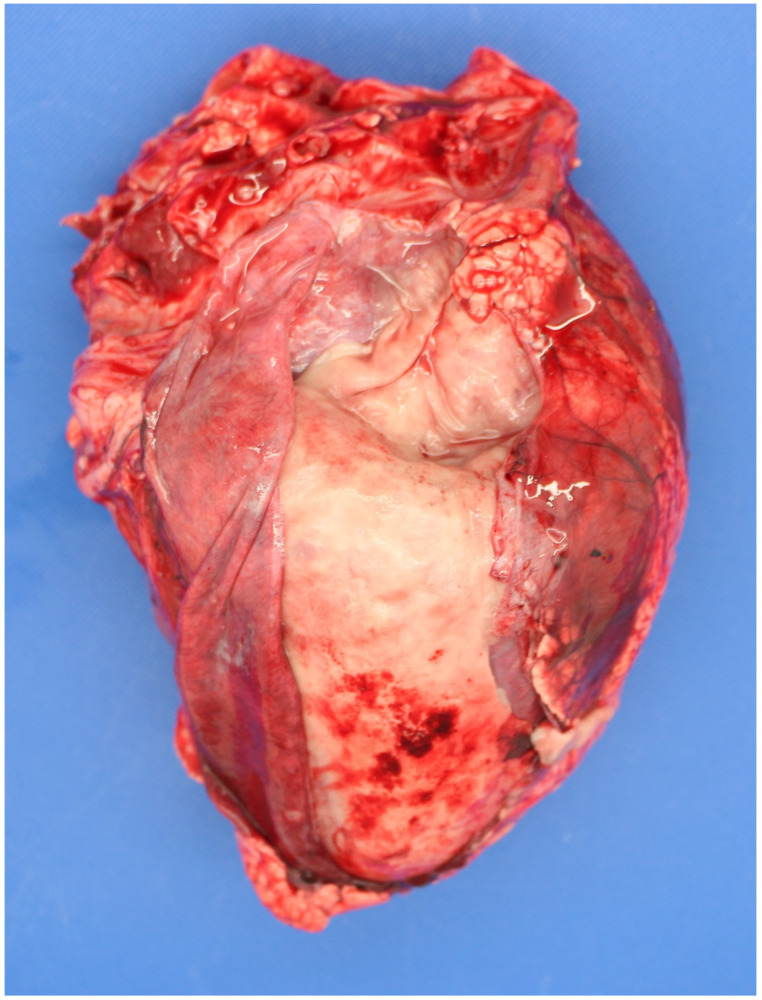
Extensive fibrinous pericarditis in a perinate infected with *Bacillus licheniformis*.

*Bacillus licheniformis* has been detected in ~≤5% of PM calves, generally in the abomasal contents, e.g., 2 and 0.8%, in studies in the UK [37] and Denmark [5], respectively. It is more commonly detected in suckler than dairy foetuses [38]. It can be more common in high risk environments, e.g., housed beef cows fed pit grass silage [39] and can cause serious loss in co-infections, e.g., BVDv [40]. *Bacillus* infection (haematogenous) typically causes necrotizing placentitis, bronchopneumonia and pericarditis, (Figure 1), [26]. Common risk factors are housed cattle offered mouldy or spoiled forage.

##### Bovine Viral Diarrhoea Virus (BVDv)

Bovine viral diarrhoea virus has generally been detected in <10% of PM cases. However, the virus can also cause serious outbreaks of PM [7] and was one of the original pathogens associated with ‘weak calf syndrome’ [41]. It is more commonly detected in suckler than dairy foetuses [38]. Virus detection rate is dependent upon diagnostic technique; antigen-detection ELISA or RT-PCR being better than IFAT, virus isolation or serology [42]. Given the low (generally <1%) prevalence of BVDv-persistently infected (PI) newborn calves internationally, unless a large number of PM calves are tested for virus it is likely that a false negative conclusion will arrived at regarding the presence of BVDv in such calves. For example, for every eight seropositive pre-colostral calves on commercial dairy farms in the USA, one PI was born [43]. However, BVDv can be detected in PM cases, e.g., in the study by Smyth et al. [23] where BVDv antigen was detected in 0.7% of PM calves. Similarly, the BVDv seroprevalence in newborn calves is low, e.g., 3.3% of PM calves in a recent Polish study [4]. This indicates that infection occurred in the later stage of pregnancy, after 125 days of gestation [44].

##### *Leptospira* spp.

*Leptospira* serovar *hardjo* has generally been detected in <10% of PM cases. The prevalence of pathogenic leptospira in PM calves may be determined by cattle population infection rates and related, use of leptospira vaccines. Hence, detection rates vary widely between countries, e.g., 3.3% PCR-positive PM cases in a recent Polish study [4] compared to 14–26% fluorescent antibody test (FAT) positive PM cases in earlier studies in Northern Ireland [23,45]. Unlike many other bacteria which are easy to culture, leptospira are difficult to detect in PM calves (e.g., the FAT to detect leptospira requires a specialized laboratory with highly skilled staff and so is being replaced by PCR, e.g., [25]. Hence, evidence of foetal exposure (foetal antibodies) is often used as a surrogate indicator of foetal infection. However, detection of low foetal titres to *Leptospira* sp. (1:100–1:200) does not exclude the involvement of these bacteria in PM. Calves experimentally infected during pregnancy with *Leptospira* serovar *hardjo* which had different outcomes (apparently viable, weak, dead) varied in the extent of their immune response (microagglutination titres from undetectable to 1:30,000 in precolostral sera) [46]. Other non-maintenance leptospira species (e.g., *L. Grippotyphosa* and *L. Australis*) have been detected in jaundiced aborted and term bovine foetuses [47].

##### *Neospora caninum* 

This parasite is commonly the most frequently detected pathogen in aborted calves (e.g., 18% in Scotland, [48] and in PM calves but infection rates are generally lower (<10%) in the latter. *Neospora caninum* is more commonly detected in dairy than suckler foetuses [38].Seropositive heifers are four-times more likely to have a PM case at first or second calving [49] and co-infection with BVDv may exacerbate this effect [50]. The percentage of PM calves with a positive humoral response (e.g., titre ≥1/320) to *N. caninum* is about 5% (4.1% in Poland [4], 5.5% in Northern Ireland, [51]. The timing of foetal infection with *N. caninum* determines the outcome of the pregnancy. Infection in late pregnancy allows the fetus to suppress infection and pregnancy ends in time for clinically normal calves to be born [52]. The highest rate of detection the *N. caninum* is in the brain, though the latter may be grossly normal. This may be because the parasite has a predilection for the central nervous system [53]. The occurrence of cell destruction, and therefore disease, depends upon the balance between tachyzoites being able to penetrate and multiply in host cells and the ability of the host to inhibit parasite multiplication [53].

#### 2.1.5. Infectious Agents Less Commonly Causally Linked to Perinatal Mortality

Numerous less common pathogens have been detected in cases of PM, in cross-sectional and longitudinal studies or often in case studies.

##### Bovine Herpes Viruses

Diagnosis of BoHV-1 and -4-associated PM is complicated by two factors. Firstly, this virus acts immunosuppressively, so foetuses whose dams were experimentally infected mount no antibody response although virus was detected in all organs (lungs, liver, spleen, kidneys, intestines, mesenteric lymph nodes) [54,55]. Secondly, many herds are vaccinated against BoHV-I and this may impact the detection rate in PM cases, e.g., in almost 30% of Polish herds cows were vaccinated against BoHV-1, which could partly explain the failure to detect BoHV-1 in PM cases from these herds [4]. Additionally, as it has been shown that BoHV-1 challenge of vaccinated cows resulted in the birth of normal calves in which BoHV-1 virus was not isolated [56]. However, in field outbreaks both abortion and PM have occurred despite vaccination [57]. Even in studies when vaccination was not widespread, the virus has rarely been detected in large scale studies of PM, e.g., in none of 293 PM cases examined by Smyth et al. [23] although it has been detected in small PM case series [55] and reported to be involved in individual outbreaks of stillbirth, e.g., [58] and experimental inoculation studies have resulted in PM [59].

##### *Chlamydia* and *Chlamydia*-Like Organisms

While *Chlamydia abortus* is a recognised cause of bovine foetopathy [60] the role of *Chlamydia*-like organisms, e.g., *Parachlamydia* spp, in the aetiology of bovine abortion and PM is unclear. For example, attempts to experimentally induce abortion with a *Waddlia chondrophilia* inoculum failed [61]. Three studies described a possible association with bovine abortions [60,62,63] but not PM. Even when detected in PM cases there may not always be associated lesions, e.g., in a recent Swiss study of the five cases of PM where these organisms were detected, three cases had inflammatory lesions (placentitis, alveolitis), while two cases were histologically normal [21]. Previous reports of PM cases in which *C. abortus* was diagnosed (by IHC) also reported placentitis [64].

##### *Coxiella burnetii* 

*Coxiella burnetii* is not commonly detected in PM studies as it has a tropism for the placenta which is often not submitted and it is not generally tested for in the foetus. However, at the herd-level, the risk of stillborn calves and perinatal death can be higher with high level of BTM antibodies [65]. While high infection rates with *C. burnetii* can be detected in PM calves (e.g., 32% of calves qPCR-positive in a recent Swiss study [21]), placental infection rate (15% [21]) and evidence of inflammatory response (placentitis and bronchopneumonia) may be much lower (e.g., 19% [21]). These findings suggest that acute infections with *Coxiella burnetii,* may occur without histological lesions. Foeto-placental infection rates reflect the infection rate in the at-risk cattle population which vary between countries based on serological surveys, e.g., 18% in Korea [66] compared to 38% in Ireland [67]. Foetal coxiellaemia associated with PM occurs sporadically in endemically infected herds and is more common in primiparae [68].

##### *Escherichia coli* 

While this is one of the most common bacteria isolated from stillborn and aborted calves [6,11], the significance of such infection is unclear; it may be an opportunist infection, a co-infection or a contaminant. One possible source of this pathogen could be an ascending infection in the reproductive tract. *E coli* is as commonly detected in suckler as in dairy foetuses [38]. *Escherichia coli* is also a common cause of postnatal (0-2 days) infectious PM—colisepticaemia [69].

##### *Fungi* and Yeasts

*Aspergillus fumigatus,* primarily [70] but also, *Asp. niger, Mucor* spp. and associated fungi and yeasts (e.g., *Candida*) are sporadically detected in PM studies, usually at a low rate (<10%). Sporadic cases predominantly occur during the winter when pregnant cows are fed on conserved mouldy forage containing saprophytic fungi [70]. In a minority of cases foetal fungal dermal plaques or a thickened placenta may be observed [70].

##### *Listeria monocytogenes* 

**Figure 2:**
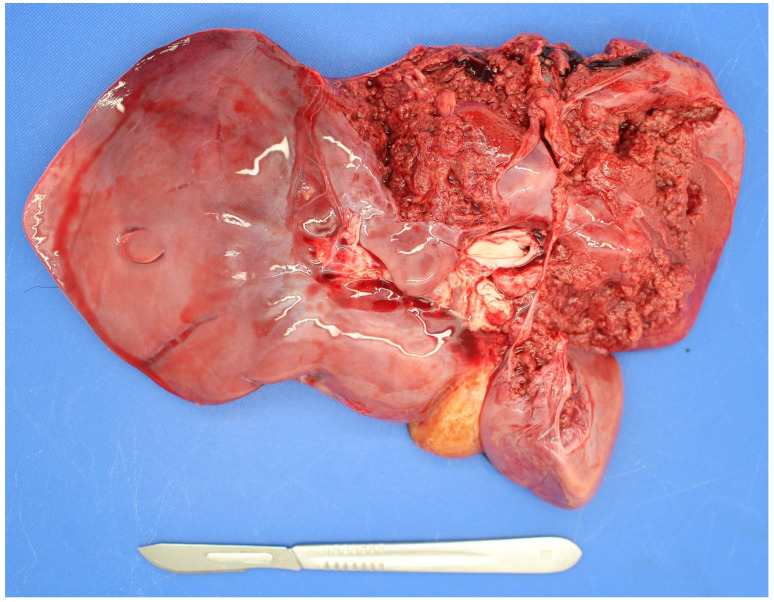
Hepatic rupture in a foetus infected with *Listeria monocytogenes*.

*Listeria monocytogenes* is not commonly detected in PM studies. It is more commonly detected in suckler than dairy foetuses [38]. As listeria spp. are soil-borne microbes they can cause infection when soil is incorporated with grass in silage pits without adequate anaerobic conditions during fermentation [71]. Both abortion and PM can result due to placentitis and foetal septicaemia with fine hepatic necrotic multifoci (sawdust liver) observed in some cases and occasionally associated hepatic rupture (Figure 2).

##### *Mycoplasma* Species

*Mycoplasma* spp. are rarely tested for and when they are, are rarely detected in PM studies, e.g., none of 121 PM cases yielded mycoplasma spp. though a small percentage of dams (5%) was seropositive [72]. They are occasionally detected in aborted foetuses [73,74,75] and experimental inoculum studies have resulted in abortion indicating they are primary pathogens [76,77].

##### *Salmonella* Species

**Figure 3:**
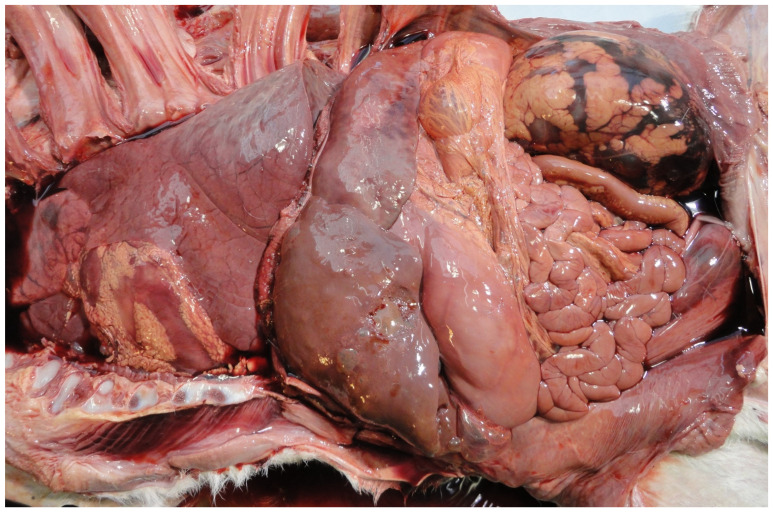
Autolysed foetus infected with *Salmonella* Dublin.

*Salmonella* species are not commonly detected in PM studies being more commonly detected in aborted foetuses [78] and associated with neonatal calf mortality [79]. The most commonly detected *Salmonella* species is *S.* Dublin [78]. Infected foetuses are often autolysed (Figure 3). *S.* Dublin is more commonly detected in dairy than in suckler foetuses [38]. However, other species, e.g., *S.* Stanley, have also been detected in cases of PM [27].

##### Schmallenberg Virus (SBV)

**Figure 4:**
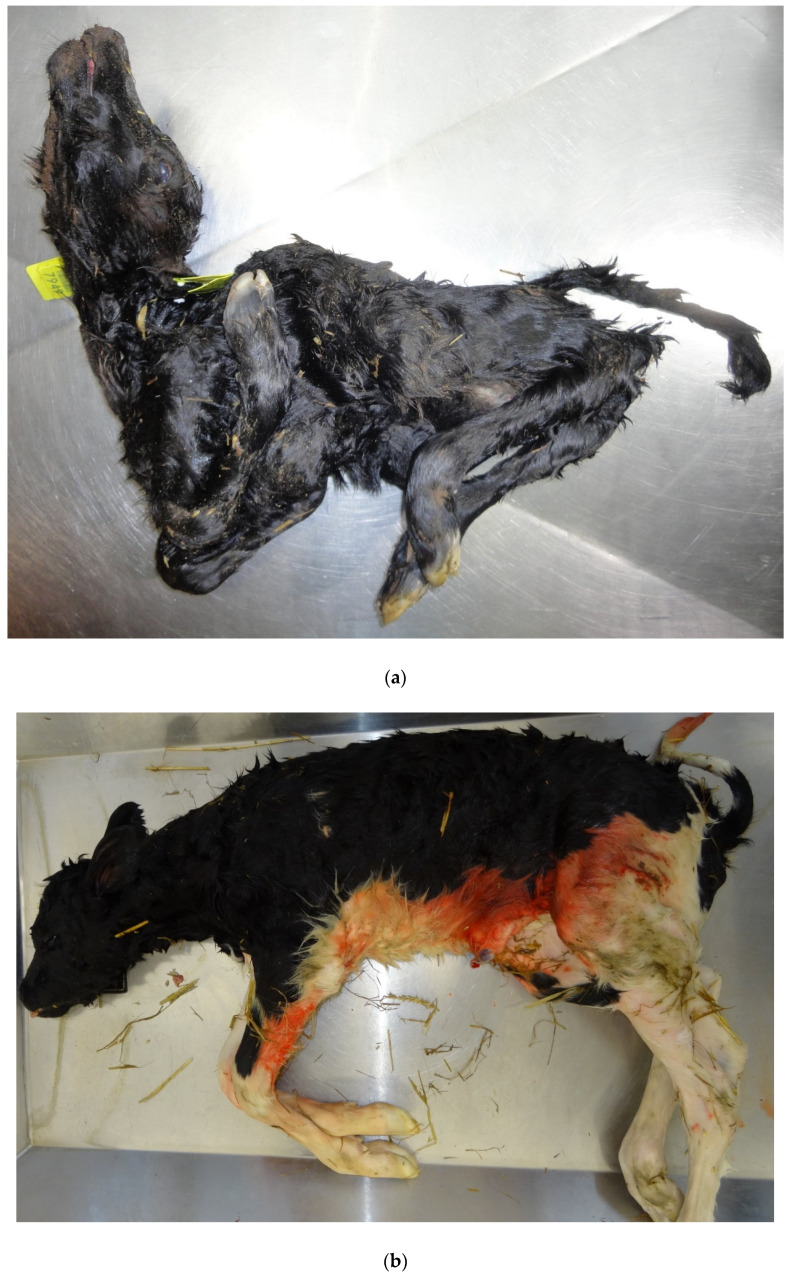
(**a**). Typical lesions of Schmallenberg infection (arthrogryposis, torticollis, kyphosis) in a bovine foetus PCR-positive for the virus. (**b**). Fetus exposed to Schmallenberg virus in utero and antibody–positive, but without the pathognomonic lesions.

This virus was first detected in cattle in 2011 and despite re-occurrences since then, it has not become a common cause of bovine foetopathy except in virus recirculation years/regions due to protective seroprevalence endemicity [8]. However, younger, naïve, populations of susceptible cattle do present the potential for future cyclic outbreaks and PM in calves. The presence of SBV antibodies in PM calves without the pathognomonic gross malformations (AHS—arthrogryposis, hydranencephaly syndrome), (Figure 4a,b), suggests infection after 47 days of gestation when the foetus is immunocompetent [8]. Most fetuses infected after immunocompetence are born alive and seropositive but without lesions indicative of SBV infection [80].

##### *Trueperella pyogenes* 

**Figure 5:**
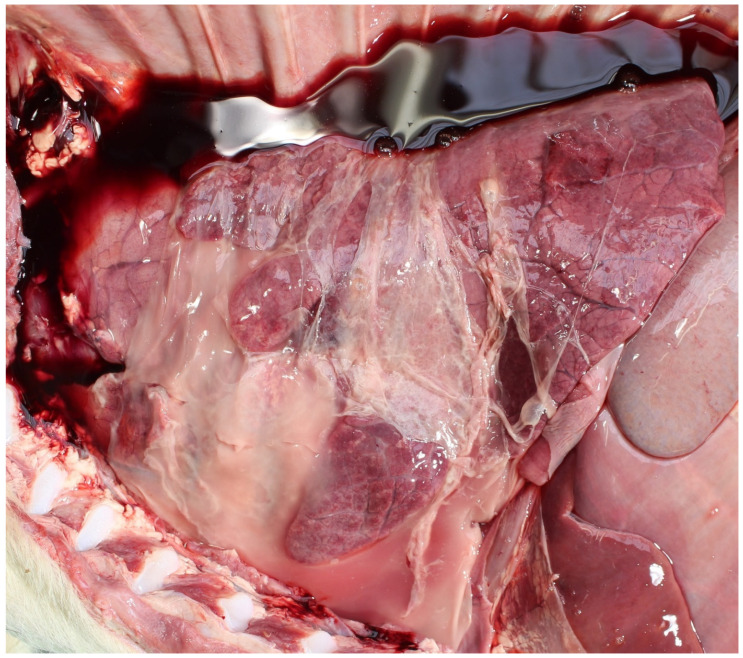
Extensive pleuropneumonia a foetus infected with *Trueperella pyogenes*.

*Trueperella pyogenes* is not commonly detected in PM studies. Perinatal mortality, usually sporadic, due to *T. pyogenes* is usually secondary to a maternal suppurative focal infection or bacteraemia, i.e., haematogenous infection [81]. The organism can cause placentitis and foetal pneumonia, pleuritis, pericarditis and/or peritonitis, often in an autolysed foetus (Figure 5). Experimental inoculation studies have shown that *T. pyogenes* (*Actinomyces pyogenes*) is a primary pathogen in pregnant cows [81].

#### 2.1.6. Contaminant Bacteria

Contaminant bacteria commonly found in samples from cases of PM include coliforms, *E. coli, Pseudomonas*, *Proteus* and mixed bacterial infections [33]. Their presence indicate autolysed or contaminated sample (e.g., scavenged carcass, environmental contamination of placenta), unhygienic sample collection, sample storage at warm ambient temperature and/or undue delay between sample collection and culture [82]. Contaminant bacteria are commonly cultured from the abomasal contents of PM cases submitted to veterinary laboratories (10-20%), but primary (5%) and secondary bacterial pathogens (<5%) are much less frequently isolated [10].

#### 2.1.7. In Utero vs. Postnatal Infection

Perinatal losses include those occurring pre-, intra- and postpartum (variably 1–2 days after calving) periods, hence, perinates may be infected in any of these periods. Given that more perinates die during and before, than after, calving (~25, 60, 15%, respectively [83]), in utero infections cause more PM than postnatal (0–2 days) infections. However, as a proportion of perinates dying in each time period (pre-, intra- and postpartum 0–2 days), more die due to infection postnatally (0–2 days): ~10, 5 and 15%, respectively [10]. In some studies in utero infections can be the first (34% of all diagnoses, [21] or second (26% of all diagnoses [14]) most common cause of PM. The types of infections also vary with time of death. Deaths which occur pre- or intra-partum attributed to infection are much more likely to be due to primary pathogens (70–75%) while deaths which occur postpartum (0–2 days) are more likely to be due to FPT and subsequent infection from ubiquitous environmental pathogens (80–90%) [10].

##### Intra-Uterine Infections

**Figure 6:**
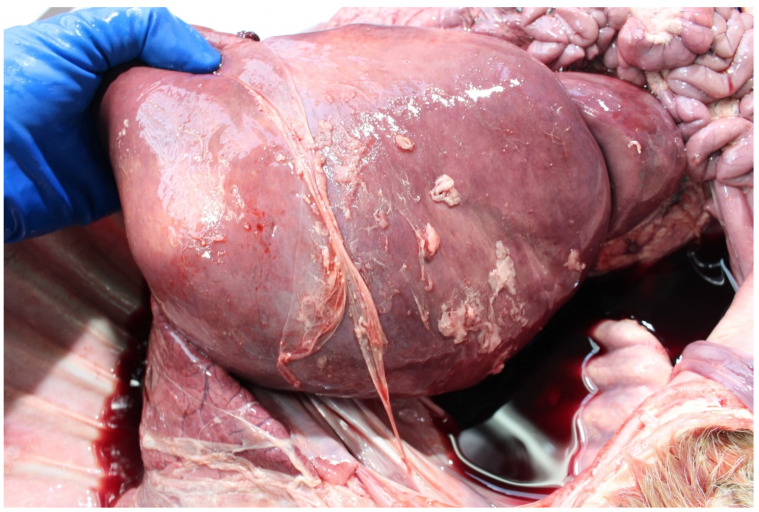
Peri-hepatic fibrinous peritonitis in a perinate which died in utero precalving.

The common sources of in utero infections are vertically transmitted transplacental infections and from the animal’s feed, water or environment [8,38,60]. Feed-borne pathogens are particularly important where feed is of poor quality, e.g., poorly fermented grass silage, more commonly in beef than in dairy herds [14] or where feed is exposed to vectors [84]. Infected foetuses may die pre-partum, during calving or postpartum (0–2 days). Common in utero infections causing PM include *Neospora caninum*, BVDv, *Bacillus licheniformis*, and *Leptospira hardjo* (see Table 3). In some cases of in utero infection no causative agent may be identified, particularly in foetuses which die in utero, and are autolysed. However, gross or histological specific (e.g., pericarditis, peritonitis and pneumonia) or non-specific findings (e.g., increased in utero B lymphocyte proliferation with germinal centre formation in the spleen indicating non-specific antigenic stimulation) indicate death due to infection (Figure 6) [85].

##### Postnatal Infections

Infection of the perinate (within two days) after birth from its new environment is normal. However, in calves with failure of passive transfer (FPT), (e.g., zinc sulphate turbidity test (ZST) result less than 12.5 units [86]) environmental infection may result in morbidity or PM [69]. Common causes include *E.coli* septicaemia (colisepticaemia), (e.g., [14]). Though their death may be predisposed by dystocia-induced asphyxia-associated FPT, farmers may be ‘blind’ to such associations or losses hence the role of postnatal infection (0–2 days) in causing PM is often underestimated [87].

#### 2.1.8. Exposure, Infection and Causality

In attempting to attribute PM to infection one must differentiate between **exposure** to infection (foetal antibody detection), the **presence of infection** (antigen detected in foetal/placental tissue) without lesions and **infectious causality** (antigen/DNA/RNA detected in foetal tissue/abomasal contents with associated lesions or the presence of compelling inflammatory lesions indicative of infection in the absence of single foetopathogen detection, e.g., pneumonia, encephalitis, pericarditis, omphalophlebitis, peritonitis, septicaemia, hepatitis, arthritis, nephritis [11] etc, and tests exclude other diagnoses). Therefore, pathogens may be detected in the presence and/or absence of lesions and lesions may be detected in the presence and/or absence of pathogens. This point is illustrated in recent findings from the placentomes of abattoir-sourced pregnant cows in which small numbers of pathogenic bacteria were isolated without the presence of associated lesions [88]. On the contrary, where there is gross or histopathological evidence of infection or response to infection, the combined case evidence may not be compelling that the presumptive foetal infection was the cause of death as opposed to being present at death. Thus detection of exposure and/or infection must be interpreted in light of other investigative findings also.

In the absence of defence mechanisms and/or a highly virulent pathogen, intrauterine infection may result in PM. The presence of antigens without an antibody response is an indicator of infection, either very early in pregnancy (before fetal immunocompetence), or later in pregnancy when the infection kills the fetus before it seroconverts [4]. The detection of pathogens at term, which infect the fetus early in gestation, requires that the agent or its genetic material persists in the fetus, which is not always the case where there is moderate-marked in utero autolysis [13]. Therefore, the detection rate and the role of infectious agents in the aetiology of PM maybe artificially low and underestimated, respectively.

These complexities highlight the difficulties in assigning a cause of death (COD) to an infectious cause. It has been recommended that evaluation of true infectious causes should focus on both antigen detection and histological examination [21].

**Figure 7:**
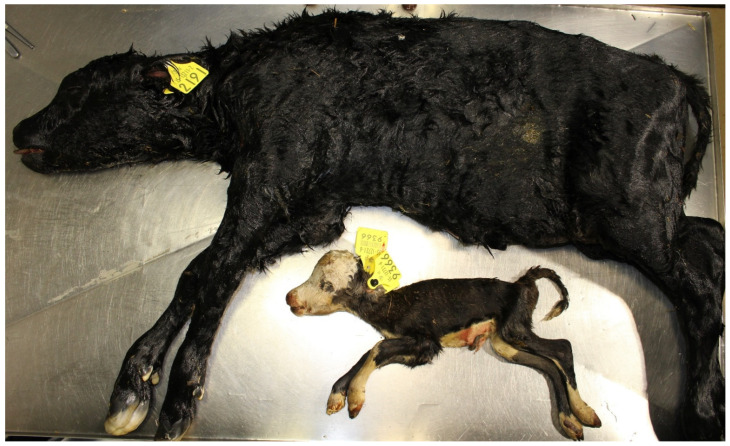
Aborted (2.4 kg, unknown gestation length) and stillborn (56.7 kg, 282 d gestation) foetuses.

In contrast to the relatively limited proportion of PM attributed to infection, in bovine abortion infection is the most important diagnosed cause of death. This disparity can be obscured as many studies report findings for both abortion and PM combined (Figure 7), (e.g., [11,12,42,89]), and depending on the terminology used to describe the foetuses it can appear that infection is a major cause of PM. For example, a Dutch study detected pathogenic antigens in 28% of aborted and stillborn fetuses [89]. Where aborted and stillborn fetus data are disaggregated, within the same study, detection of pathogenic antigens is always significantly lower in stillborn calves. For example, at the lower end, a Canadian study diagnosed an infectious cause of death in 16% of aborted and in only 3% of stillborn fetuses [13] while at the higher end, a Finnish study detected significant bacterial isolates in 26 and 10% of aborted fetuses and stillborn calves, respectively [6]. These studies indicate that at term non-infectious causes of death, e.g., dystocia and asphyxia, are more important than infectious causes of death.

#### 2.1.9. Proportion of Perinatal Mortality Caused by Infection

The proportion of PM attributed to infection can vary tenfold between studies: ~3 to 35% (Table 1). Some common reasons for high variability in the attributable fraction of PM caused by infection include conflation of data from aborted and stillborn calves, geographic differences in infection prevalence and control methods (e.g., vaccination, eradication), differences in definition of ‘infection’, and differences in sampling protocols.

Although there are many reports investigating infection as a cause of abortion (e.g., [82]), few focus on the role of infectious agents in PM specifically and in some cases results for aborted and PM calves are combined (e.g., [89]). Unlike abortions where infections constitute the major proportion of diagnosed causes, in perinatal mortality infections are a minor diagnosed cause, generally less than 15% (with exceptions) in published studies (Table 1). However, in high mortality herds in regions where particular infections are endemic, the proportion of perinatal mortality attributable to infections may be much higher (e.g., [21]). Hence, one cannot cite data on infection rates and types in the international literature without reference to the biosecurity conditions in the country in which the data were collected. Thus, in countries free from specific infections, e.g., *Brucella abortus* (Poland, Republic of Ireland, Switzerland), Bovine Herpes Virus-I (BHV-I), (Sweden, Finland) or pathogenic *Leptospira* spp. (Sweden) [6,20]), those infectious agents will not be detected in PM calves.

Infectious foetopathy diagnostic rates in cases of PM also vary between studies depending on whether detection of exposure to infection (foetal antibody positive) or the presence of infection (antigen detected in foetal/placental tissue) with/without lesions, is the diagnostic criterion [4,21,90], as discussed previously. Compelling inflammatory lesions indicative of bacterial infection (e.g., bronchopneumonia, encephalitis, pericarditis, hepatitis, omphalophlebitis and peritonitis) are often ranked as the most commonly detected criterion [13,14,31]. Variation in sampling protocols may also account for apparent differences in the proportion of PM cases caused by different pathogens. For example, in some studies all calves are tested, irrespective of gross pathology [4], while in others, only tissues with macroscopic pathological lesions are sampled [5]. These protocol differences may account for a lower infection detection rate in studies where selective sampling is implemented and a relatively higher detection rate in studies where all calves are sampled.

## 3. Diagnosis of Infectious Causes of Perinatal Mortality

The current state-of-the-art approach to investigation of all-cause perinatal mortality in calves has recently been reviewed [91]. The possible novel diagnostic approaches of the future are presented by Jawor et.al. [2] in Part 2 of this mini-series. The standard operating protocol (SOP) involves three steps: collect a history, examine the pregnant animals and the dam and examine the foetus/placenta. The focus here is on diagnosis of infectious causes only. While the criteria used to diagnose infection as a cause of PM are generally agreed, there are many caveats to the details of such attributions [1]. As with bovine abortions, the more sample material examined (i.e., foetus, and placenta and maternal blood), the higher the likelihood of an aetiological diagnosis [92].

### 3.1. Herd Health History

In the context of possible infectious causes, the herd health history is important. For example, details of most recent vaccinations against foetopathogens, recent cattle purchases, grazing cows on out-farms (e.g., alpine pastures), introducing heifers, raised on contract-rearing farms, to “home farm” before calving and (antibody/antigen) bulk milk test results.

### 3.2. Cohort Sampling

Examining the pregnant cohort and their environment allows assessment of general herd health. Cohort sampling of dams (sero-diagnosis) in the affected group can be useful in determining differences in exposure (presence/absence, prevalence) between affected and unaffected dams (case–control), but vaccinal status needs to be known [91].

### 3.3. Examination of the Dam

Clinical examination of the dam may reveal signs of infection, e.g., pyrexia, diarrhoea, respiratory signs, etc. A faecal sample may be useful where salmonellosis is suspected [91]. A single blood sample from the affected dam can have moderate utility as a proxy sample for foetal material. For example, a single blood sample from the non-vaccinated dam of a stillborn or aborted foetus can be up to 85% accurate in predicting a foetal culture-positive result for *Salmonella* Dublin [78]. However, the primary value of maternal serum is as an exclusionary test for maternal antibody. For example, marked rises in maternal *Neospora caninum* antibody titres occur during the second half of gestation associated with vertical transmission in experimental and field studies [93]. Hence, seronegative fullterm dams are unlikely to deliver a *Neospora*-positive foetus. However, repeat sampling may be necessary to rule out those cases whose antibody status fluctuates between seronegative and seropositive (false negatives): ‘serologically elusive animals’ [93]. Paired maternal sera (more than two weeks apart) may detect increased antibody values (e.g., two to four-fold titre rise, + to ++++, or increased absorbance) for some foetopathogens (e.g., *S.* Dublin SAT) but not for others (e.g., *Leptospira* spp., *Neospora*) due to the lag phase between infection and foetal mortality [78,91]. Higher positive rates in maternal serology than in foetal antigen detection is not uncommon given sero-endemicity with common pathogens, e.g., Vidal et al. [25]. In vaccinated herds natural infection can still be distinguished where DIVA vaccines are used (gE-deleted BoHV-I) and where maternal titres are much higher than those expected from vaccination (e.g., *Leptospira*) this suggests current, active infection [91].

### 3.4. Examination of the Foetus

This examination involves three steps—external and internal examination and sampling the carcass as detailed in a recent illustrated guide to bovine foetopsy [94]. In order to avoid artefactual changes in the carcass and placenta, they should be stored in away from animal access. The external examination may detect foetal undersize (low body weight and/or short CRL), a possible indicator of foetal infection and shortened gestation [4]. Internal examination may reveal gross evidence of infection (which may or may not be confirmed by laboratory analyses). One of the most common lesions is pneumonia, from prenatal infection (e.g., pathogen aspiration from amniotic fluid or haematogenous spread), intranatal hypoxia (e.g., meconium aspiration possibly induced by placentitis) or postnatal (0–2 days) infection (e.g., colostrum aspiration pneumonia from misuse of an oro-gastric feeder or perinatal septicaemia) [10,85]. Other lesions include pericarditis, meningitis, hypopyon, intestinal rupture, DIC with haemorrhages and sepsis [91]. It is recommended that once the carcass is opened samples for microbiological testing are collected first to avoid contamination during the internal organ examination [91].

#### 3.4.1. Sampling the Carcass

**Figure 8:**
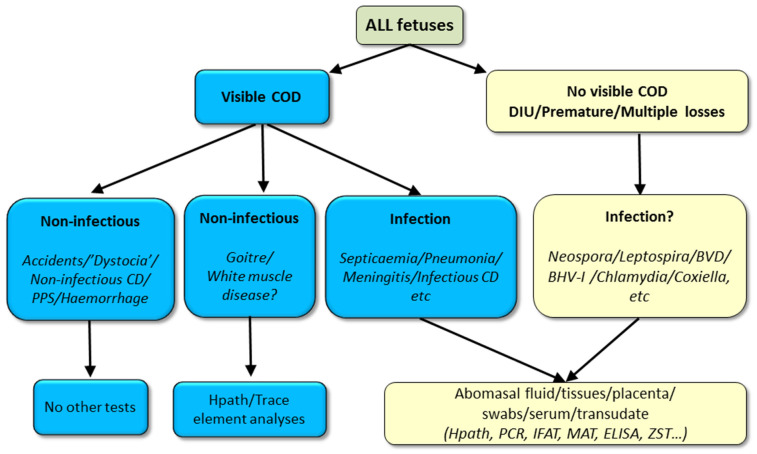
Necropsy sample selection decision tree for stillborn foetuses (COD = cause-of-death, CD = congenital defect, DIU = dead in utero). Footnote. While a non-infectious cause of death may be grossly suspected or apparent, occult infection may also be present, hence, where mandated by law or where infection is also suspected, sample/s should be collected to rule out infection, typically, abomasal contents.

A sample selection algorithm for stillborn foetuses is outlined in Figure 8. It is not possible to be prescriptive about test selection as laboratories differ in the test menu and tiers they offer. Test selection will also be determined by the anamnesis, degree of carcass autolysis and costs. Rather than ‘necropsy by algorithm’ the decision-making of the veterinary practitioner or pathologist still determines whether and which samples to collect. Additionally, samples can be discarded if collected in the early stages of the necropsy where subsequent examination reveals the likely cause does not require laboratory testing for infectious causes [91].

##### *Microbiology* Samples

Abomasal contents (for culture, PCR and microscopy) are amongst the most important sample to collect as they represent the amniotic fluid in which the foetus lives throughout pregnancy [91]. They can be sampled aseptically by searing the abomasal serosa with a heated scalpel blade and aspirating a sample into a plain vaccutainer tube. If an abomasal sample is unobtainable (due to scavenging or colostrum ingestion) or if septicaemia is suspected, lung, liver or brain samples are suitable alternatives, though not as sensitive [33]. In general, tissue samples are preferable to swabbed samples and surface swabbed samples are preferable to fluid swab samples [91]. Brain sampling is of particular value in severely scavenged and mummified foetuses. The presences of a pure growth of a pathogen with associated lesions, in the absence of other causes of death, are usually accepted as diagnostic criteria.

#### 3.4.2. Serology Samples

Serological sampling of the foetus can be useful from the second trimester (>120 days—pathogen-dependent) when the foetus is immunocompetent as antibodies indicate foetal infection (but not necessarily foetopathy, e.g., *Neopsora* congenital infection), assuming placental competence [91]. However, reliance on foetal serology alone may grossly underestimate foetal infection rates [90]. The details on these and other biomarkers are discussed by Jawor et al., [2] in Part 2 of this mini-series.

#### 3.4.3. Histopathology Samples

Tissues with gross lesions indicative of infection plus lung, liver, heart and brain are routinely collected for histopatholoical examination [95]. The brain is of particular value in the histopathological diagnosis of neosporosis. Samples for histopathology should include normal and adjoining abnormal tissue and should not be greater than 1 cm thick and 2 cm long as they need to fit into processing cassettes (3.5 × 2.5 × 0.5 cm) [91].

#### 3.4.4. Standard Microbiology Package

Some microbiological tests are carried out routinely on all PM cases in veterinary diagnostic laboratories (‘standard microbiology package’) while other tests are non-routine (e.g., PCR, histopathology) [91]. While for sporadic cases the basic sampling package may suffice (Table 4), in PM outbreaks, tiered sample escalation to an extended spectrum analysis is advised as it provides the option of sampling, storing and testing as deemed necessary.

#### 3.4.5. Sample Storage

If microbiology samples are collected on a day when the veterinary laboratory is closed, e.g., weekends, holidays, the samples should be stored for culture, serology and histopathology in a fridge (4 °C) and those for PCR in a freezer (−20 °C) [91].

### 3.5. Examination of the Placenta

**Figure 9:**
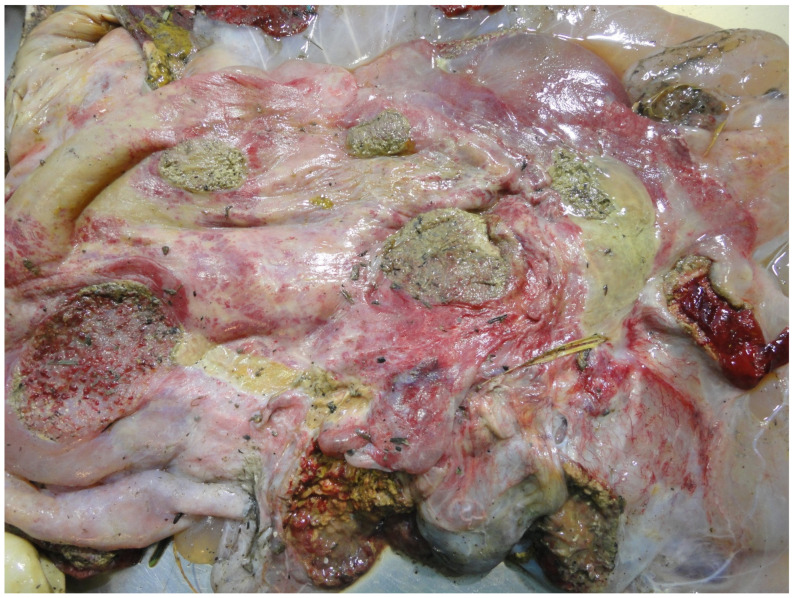
Severe cotyledonary and intercotyledonary placentitis.

If the placenta has not yet been expelled at examination, a sample of the retained placentomes or a vaginal swab can be obtained [91]. A normal placenta at term weighs approximately 5 kg, has 75–125 red cotyledons and has thin, translucent intercotyledonary tissue that sometimes contains adventitious placentation [91]. Placentitis may be grossly manifest as discoloured, necrotic, exudative cotyledons and opaque, erythematous, thickened, oedematous intercotyledonary tissue (Figure 9) [96]. Ideally three abnormal cotyledons/inter-cotyledonary tissue samples should be submitted (preferably macroscopically abnormal and uncontaminated) for histopathological and microbiological examination (Gram smear, modified Ziel Neelsen, culture, PCR) [91]. Sampling of the placenta is of particular importance where *Coxiella burnetii* or *Chlamydia* spp. are suspected [21]. Two of the major problems with placental diagnostics is unavailability; in only a minority of cases (<10%) of PM does the placenta accompany the foetus and environmental contamination of the temporary organ [10].

## 4. Conclusions

One of the most important outcomes from this review is that the diagnosis rates of infection as a cause of PM are highly variable between studies; this indicates a need for standardisation of diagnostic approaches. This review has also identified that most of the important infectious causes of PM can be controlled by management; better pregnant cow feed conservation/storage (e.g., *Bacillus licheniformis*), vaccination (e.g., *Leptospira hardjo*, BVDv) and biosecurity (e.g., *Neospora caninum*). While the importance of in utero infections as a cause of PM is generally recognised, the role of postnatal (0–2 days) infection, often predisposed by failure of passive transfer, can be underestimated. This differentiation is relevant to their disparate control measures. Necropsy examination is central to the current approach to diagnosis of infectious causes of PM but non-submission of the placenta often hampers a comprehensive investigation.

## Figures and Tables

**Table 1:** Necropsy-diagnosed causes-of-death (%) for calves dying in the perinatal period (0–48 h) internationally.

Country	Perinate Definition	Calves (No.)	Calf Type	Dystocia	Anoxia	Congenital Defects	Infection	Other	Unknown	Reference
Argentina	Gestation >260 d—stillborn	19	Dairy	21.1	NR ^1^	NR	10.6	5.3	68.4	[9]
Belgium	Gestation fullterm—died ≤24 h	NR	Dairy & Beef	7	55	6	18	4	10	[15]
Canada	Gestation >8 mths—died <1 h	560	Beef	40.2	NR	4.3	4.3	31	20.2	[13]
Denmark	Stillbirth	130	Dairy & Beef	9	81	1.5	8	0.1	0	[5]
Finland	Gestation fullterm—died ≤24 h	148	Dairy	43	NR ^2^	10	14	8	25	[6]
Iceland	Gestation fullterm—died ≤24 h	129	Dairy	34	37	NR	12	13	3.9	[16]
Ireland	Gestation ≥260 d—died ≤48 h	1345	Dairy	33	10	11	9	28	9	[10]
Japan	Stillbirth	155	Beef	21	NR	3.9	NR	5.1	69.7	[17]
Netherlands	Stillbirth	193	Dairy	4	37	8.3	7.3	6	42	[18]
Nr. Ireland	Gestation fullterm—died ≤10 min.	365	Dairy	23	46	NR	31	NR	NR	[19]
Poland	Gestation fullterm—died ≤6 h	121	Dairy	NR	NR	NR	9.9	NR	NR	[4]
Scotland	Gestation fullterm—died ≤48 h	54	Beef	37 ^2^	NR	11.1	35.2	3.7	13.0	[14]
Sweden	Gestation fullterm—died ≤24 h	76	Dairy	46.1	NR	5.3	2.6	10.5	35.5	[20]
Switzerland ^3^	Gestation ≥260 d—died ≤48 h	47	Dairy	4.3	NR	0	34	4.2	57.5	[21]
USA	Gestation fullterm—died ≤48 h	60	Dairy	25	28.5	3.3	5	6.6	31.6	[22]

^1^ NR = not recorded, ^2^ anoxic and dystocic lesions combined, ^3^ Ultimate cause of death (COD).

**Table 2:** List of primary and secondary pathogens associated with bovine foetopathy in alphabetical order *.

Primary	Secondary
*Akabane virus*	*Absidia* spp.
*Anaplasma phagocytophilium*	*Arcobacteria* spp.
*Aspergillus* spp.	*Acinetobacter species*
Bluetongue virus	*Actinomyces* spp.
*Brucella abortus*	*Aerococcus* spp.
Bovine adenovirus 5	*Aeromonas hydrophila*
Bovine herpes viruses 1 and 4	*Bacillus* spp.
Bovine viral diarrhoea virus	*Chlamydia-related organisms (CRO)*, e.g., *Parachlamydia-like organisms (PCO), Waddlia* spp.
*Bacillus licheniformis*	*Citrobacter youngae*
*Campylobacter foetus* spp.	*Escherichia* spp.
*Chlamydophila abortus*	*Fusobacterium spp*
*Clostridium perfringens*	*Hafnia alvei*
*Coxiella burntiii*	*Histophilus somni*
*Leptospira* spp.	*Klebsiella pneumonia*
*Listeria monocytogenes*	*Listeria* spp.
*Mycoplasma* spp.	*Mannheimia* spp.
*Neospora caninum*	*Neisseria,*
*Parainfluenza 3 virus*	*Nocardia* spp.
*Salmonella* spp.	*Pantoea agglomerans*
*Schmallenberg virus*	*Pasteurella* spp.
*Trichomonas foetus foetus*	*Providencia stuartii*
*Trueperella pyogenes*	*Serratia* spp.
	*Staphylococcus* spp.
	*Streptococcus* spp.
	*Ureaplasma* spp.
	Yeasts
	*Yersinia* spp.

* Readers are referred to standard veterinary microbiology textbooks for further information on these microbes.

**Table 3:** Distribution of pathogens (antigens) detected in calves dying in the perinatal period (0–48 h) internationally (% of dead perinates).

Country	Infected Calves (No.)	Infection(%)	*Bac. lich.*	*Cox. burn.*	Fungi	*Lepto.* spp.	*List.* spp.	*Neo. cani.*	*Tru. pyog.*	*Salm.* spp.	Viruses	Other inf.	Reference
Argentina	19	10.6	NR	NR	NR	NR	NR	5.3	NR	NR	NR	5.3	[9]
Belgium	NR	18	NR	NR	NR	NR	NR	NR	NR	NR	12	6	[15]
Canada	560	4.3	NR	NR	NR	NR	0.4	0.2	NR	NR	0.2	3.5	[13]
Denmark	130	8	1	NR	NR	NR	NR	NR	NR	NR	7	NR	[5]
Finland	148	14	5	NR	NR	NR	NR	2	0.7	NR	NR	6.3	[6]
Iceland	129	12	NR	NR	NR	NR	NR	NR	NR	NR	NR	12	[16]
Ireland	1345	9	0.80	NR	NR	0.6	0.2	1.5	0.9	0.2	0.8	4	[10]
Netherlands	193	7.3	1.6	NR	NR	NR	NR	NR	NR	1.6	4.1	NR	[18]
Nr. Ireland	365	31	NR	NR	NR	13.4	NR	NR	NR	NR	NR	17.6	[19]
Poland	121	9.9	NR	NR	NR	NR	NR	7.4	NR	NR	NR	1.7	[4]
Scotland	54	35.2	5.5	NR	3.7	1.9	NR	NR	NR	NR	NR	24.1	[14]
Sweden	76	2.6	NR	NR	NR	NR	NR	NR	NR	NR	NR	2.6	[20]
Switzerland	47	77	NR	31.9	8.5	6.4	NR	2.1	NR	NR	NR	28.1	[21]
USA	60	5	NR	NR	NR	NR	NR	NR	NR	NR	5	NR	[22]

Co-infections are listed under ‘Other infection’, NR—not reported.

**Table 4:** Standard and additional samples to collect from cases of perinatal mortality * for the investigation of infectious causes of death.

For Investigation of	Standard Samples	Ancillary Samples	Comments
Failure of passive transfer of immunoglobulins	Perinate blood	NA **	Only test calves >24 h old, e.g., ZST test
Foetopathogenic bacteria and fungi (e.g., *Aspergillus* spp., *B. licheniformis, L. monocytogenes, T. pyogenes*, *S.* Dublin)	Foetal stomach contents (FSC), Placenta (ideally collected from dam to reduce contamination)	Foetal lung, liver, gall bladder, kidney, brain, eyelid. Dam vaginal swab, placentome, blood.	Ancillary samples where FSC/placenta unavailable/contaminated.
*Neospora caninum*	Foetal brain, serum	Foetal heart. Placenta. Dam/cohort bloods	Fresh brain/placenta for PCR, fixed brain or heart/placenta for histopathology if PCR positive
*Leptospira Hardjo*	Foetal kidney, serum	Dam/cohort bloods	Foetal sample dependent upon lab. tests
BVDv	Foetal ear, spleen, thymus, serum	Foetal kidney. Dam/cohort bloods	Foetal sample dependent upon lab. Tests
BHV-I	Foetal liver, serum.	Foetal kidney. Placenta. Dam/cohort bloods	Foetal PCR/histopathology preferred tests
Gross lesions (e.g., foetal pneumonia)	Affected foetal organ	As required	As appropriate (e.g., bacteriology, histopath)

* Standard and ancillary testing protocols are dependent upon local laboratory SOPs. Bacteriology/mycology (culture, stains, wet preparations) and serology are generally routine tests for sporadic cases while other tests (e.g., histopathology, PCR, FAT, IHC, micronutrient, DNA assay) can be added for multiple losses or at the discretion of the pathologist. Maternal vaccinal status affects use and choice of serology tests, ** NA—not applicable.

## Data Availability

No new data were created or analyzed in this study. Data sharing is not applicable to this article.
